# Surveying the Role of Analytics in Evaluating Digital Mental Health Interventions for Transition-Aged Youth: Scoping Review

**DOI:** 10.2196/15942

**Published:** 2020-06-25

**Authors:** Brian Lo, Jenny Shi, Elisa Hollenberg, Alexxa Abi-Jaoudé, Andrew Johnson, David Wiljer

**Affiliations:** 1 Office of Education Centre for Addiction and Mental Health Toronto, ON Canada; 2 Information Management Group Centre for Addiction and Mental Health Toronto, ON Canada; 3 Institute of Health Policy, Management and Evaluation University of Toronto Toronto, ON Canada; 4 Education, Technology and Innovation University Health Network Toronto, ON Canada; 5 Department of Psychiatry Faculty of Medicine University of Toronto Toronto, ON Canada

**Keywords:** user engagement, mobile apps, mHealth, telemedicine, mental health, adolescent, data analytics

## Abstract

**Background:**

Consumer-facing digital health interventions provide a promising avenue to bridge gaps in mental health care delivery. To evaluate these interventions, understanding how the target population uses a solution is critical to the overall validity and reliability of the evaluation. As a result, usage data (analytics) can provide a proxy for evaluating the engagement of a solution. However, there is paucity of guidance on how usage data or analytics should be used to assess and evaluate digital mental health interventions.

**Objective:**

This review aimed to examine how usage data are collected and analyzed in evaluations of mental health mobile apps for transition-aged youth (15-29 years).

**Methods:**

A scoping review was conducted using the Arksey and O’Malley framework. A systematic search was conducted on 5 journal databases using keywords related to usage and engagement, mental health apps, and evaluation. A total of 1784 papers from 2008 to 2019 were identified and screened to ensure that they included analytics and evaluated a mental health app for transition-aged youth. After full-text screening, 49 papers were included in the analysis.

**Results:**

Of the 49 papers included in the analysis, 40 unique digital mental health innovations were evaluated, and about 80% (39/49) of the papers were published over the past 6 years. About 80% involved a randomized controlled trial and evaluated apps with information delivery features. There were heterogeneous findings in the concept that analytics was ascribed to, with the top 3 being engagement, adherence, and acceptability. There was also a significant spread in the number of metrics collected by each study, with 35% (17/49) of the papers collecting only 1 metric and 29% (14/49) collecting 4 or more analytic metrics. The number of modules completed, the session duration, and the number of log ins were the most common usage metrics collected.

**Conclusions:**

This review of current literature identified significant variability and heterogeneity in using analytics to evaluate digital mental health interventions for transition-aged youth. The large proportion of publications from the last 6 years suggests that user analytics is increasingly being integrated into the evaluation of these apps. Numerous gaps related to selecting appropriate and relevant metrics and defining successful or high levels of engagement have been identified for future exploration. Although long-term use or adoption is an important precursor to realizing the expected benefits of an app, few studies have examined this issue. Researchers would benefit from clarification and guidance on how to measure and analyze app usage in terms of evaluating digital mental health interventions for transition-aged youth. Given the established role of adoption in the success of health information technologies, understanding how to abstract and analyze user adoption for consumer digital mental health apps is also an emerging priority.

## Introduction

### Background

Transition-aged youth, youth between the ages of 15 and 29 years, may face difficult challenges during the transition from childhood to adulthood [1]. The ongoing challenges to provide adequate support for transition-aged youth are reflected by the observed increase in child and adolescent mental health problems [2-5]. In fact, over half of all mental health disorders among adults begin during childhood and adolescence [6,7]. And currently, mental health and substance use disorders are responsible for 25% of all years lived with disability [8]. In Canada, 11% of youth aged 15-24 years reported experiencing symptoms of depression, and 14% reported experiencing suicidal thoughts in the past [9]. These issues are further complicated by the ongoing stigma related to mental health issues [10,11] and the lower likelihood among this population to seek help [12-14]. Novel approaches to support the mental health needs and demands of this population are warranted to address these challenges [15].

### Mobile Health Interventions

Mobile health (mHealth) interventions have been identified as a promising avenue to bridge the gap between seeking help and accessing mental health resources for the youth [16-18]. The youth are well acquainted with the use of technology [18], and mobile phone use is deeply embedded in their daily lives [19]. In addition, qualitative interviews with youth show positive perceptions of mHealth interventions for mental health needs [20]. Reviews on digital mHealth interventions for youth in general [21] and college students as a group [22] have suggested that mHealth interventions can be powerful platforms for improving the overall well-being or enhancing mental health treatments among this population. The promising effectiveness of mHealth as a means of delivering mental health interventions among the youth has led to the proliferation of digital technologies.

Despite these advances, the proliferation of and interest in youth-oriented mental health apps do not always directly translate to real-world outcomes. Numerous explanations exist, and a major barrier identified by Torous et al [23] is the low engagement or uptake of these digital interventions. Since engagement is often considered a prelude to the effectiveness of digital interventions, this warrants a closer examination of how end user engagement is being measured across evaluations of youth-targeted mHealth interventions. [24]. As highlighted in a recent review of this area by Pham et al [25], the unique challenges in measuring and evaluating engagement are not limited to the heterogeneous terms used in reporting engagement levels (ie, adherence, usage, feasibility, adoption, and activity) but extend to the depth and breadth of analytics metrics being selected for measurement. This creates difficulty in selecting, interpreting, comparing, and aggregating data on engagement metrics related to these youth-targeted mHealth interventions. To determine the current state of engagement reporting and inform future efforts, we performed a scoping review of analytic metrics that were measured, reported, and used to inform the evaluation of youth-targeted digital mental health interventions. This study was used to inform the development of a randomized controlled trial (RCT) to evaluate Thought Spot [26-29], a mobile app designed to foster mental health and wellness help-seeking in transition-aged youth across the Greater Toronto Area.

## Methods

### Overview

We conducted a scoping review using the framework proposed by Arksey and O’Malley [30], which consists of 5 main processes: (1) identifying the research question, (2) identifying relevant papers, (3) selecting studies, (4) extracting the data, and (5) collating and summarizing the data and results.

### Identifying the Research Question

The main objective of this scoping review was to explore how analytics metrics are measured, reported, and used to evaluate mHealth interventions that target mental health–related issues among transition-aged youth. Under this overarching main research objective, we sought to answer the following 2 research questions to guide the review and analysis:

1. Which analytics metrics are used in evaluation studies for mHealth interventions that target transition-aged youth?
2. How do user activity and usage metrics contribute to the interpretation of study data?

### Identifying Relevant Papers

A search strategy was developed in consultation with a specialist librarian and the research team. A search using key terms such as adoption, evaluation, mental health, transition-aged youth, and mHealth was conducted in July 2018, from 5 databases: Cumulative Index of Nursing and Allied Health Literature (CINAHL), EMBASE, Medical Literature Analysis and Retrieval System (MEDLINE), PsycINFO, and Cochrane Central Register of Controlled Trials (CENTRAL). The full-search strategy for one of the databases (MEDLINE) is presented in Multimedia Appendix 1. Given the timeline of mHealth proliferation, it was deemed adequate to include only papers from the past 10 years (2008-2018) in the initial search [31]. All peer-reviewed literature was included in the review. The search was updated in June 2019, and additional papers were included in the analysis.

### Selecting Studies

After removal of duplicate papers, titles and abstracts were screened independently by 2 authors (BL and JS). There were 3 inclusion criteria: the papers examined mental health mHealth interventions designed for transition-aged youth (aged 15-29 years), included an analysis of engagement or user activity metrics, and published in English. For studies that did not indicate a specific age range, the mean and SD of the sample were used to determine the eligibility for inclusion. Studies containing only self-reported usage data or those that were developed for clinician use only were excluded, along with reviews, conference reports, and dissertations. No restrictions were placed on the type of research design or the method of comparison being made to ensure that a broad selection of studies were captured for this review.

Inter-rater reliability in screening by both reviewers was enhanced by first conducting a pilot screen of 100 papers. Pilot screens were completed independently until a satisfactory kappa statistic >0.7 [32] was reached. Two reviewers then conducted title and abstract screening independently for all papers using Abstrackr (Center for Evidence Synthesis in Health, Brown University) [33]. Any discrepancies were resolved through discussion, resulting in a consensus.

### Extracting Data

A data extraction form adapted from the Cochrane data extraction template [34] was used to extract data from each paper. A summary of the data extracted from each paper is given in Multimedia Appendix 2. This process was also performed independently by 2 authors (BL and JS) to ensure that all relevant data were accurately captured. The data were then collated into a spreadsheet for further analysis. Extracted study details included study design (eg, RCT, observational), sample size, participant characteristics, targeted condition, main research question, and primary and secondary outcomes. The target condition was extracted and categorized using the 5th edition of the *Diagnostic and Statistical Manual of Mental Disorders* [35], and interventions were classified using the *Classification of Digital Health Interventions* [36] from the World Health Organization (WHO). Additional data were also gathered based on the research questions outlined above, such as usage metrics, terminology, results, and method of analysis.

### Summarizing the Data

Quantitative and qualitative analyses of data were conducted. Descriptive statistics (eg, means, medians) were collected and used to characterize and describe each included paper. We identified the metrics of our included papers using a similar approach to that of another recently published review [25]. The themes were reviewed and extracted using a content analysis approach [37] to address the second research question. Two members of the research team (BL and JS) reviewed the data and ensured comprehensiveness and accuracy.

## Results

### Selection of Included Studies

**Figure 1:**
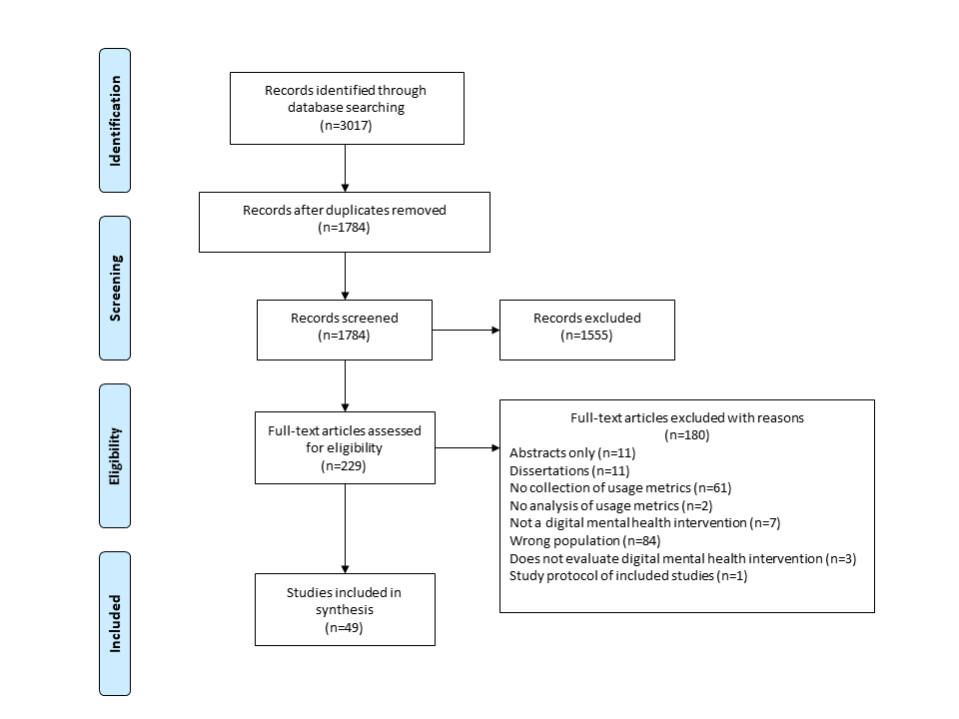
Preferred Reporting Items for Systematic Reviews and Meta-Analyses diagram of included studies.

An overview of the selection of included studies is presented using the Preferred Reporting Items for Systematic Reviews and Meta-Analyses [38] diagram in Figure 1. The database search identified 1784 nonduplicated papers. The kappa statistic, a measure of inter-rater reliability, for the pilot screen was 0.72, meeting the above-defined threshold. Following the title and the abstract review, 229 papers were fully reviewed and assessed based on the inclusion and exclusion criteria. This full-text screening was conducted independently by 2 authors (BL and JS).

### Characteristics of Included Papers

The characteristics of and references to the included studies are described in Table 1. Of the 49 papers included in this scoping review, there were 40 full reports that collected and reported usage data and 9 protocol papers that indicated an analysis of usage data in the evaluation. The findings identified 40 unique digital mental health interventions that were evaluated. Although the search strategy included studies published between 2008 and 2019, 80% (39/49) of the studies identified for inclusion were published within the last 6 years (2014-2019). The largest number of studies were conducted in the United States (21/49) and Australia (12/49).

A large number of studies have examined the efficacy or effectiveness [39-69] of the intervention in supporting individuals (eg, symptoms) as their primary objective. Studies that included other constructs (eg, feasibility [51,66,70-73], acceptability [51,57,66,69-75]) as primary objectives were typically in the earlier stages of development. In terms of methodology, the majority of studies (38/49) used or intended to use RCT methodology to conduct their evaluation. The remaining 11 studies used quasi-experimental approaches, including observational studies and single-group pretest posttest design. Studies using quasi-experimental approaches seem to be in the earlier stages of development. The sample size (or expected sample size) had a median of 100 participants and ranged from 10 to 8242 participants. Approximately 51% (25/49) of the included studies had up to 100 participants for analysis. The length of exposure to the intervention was also captured, and 47 of the 49 studies reported the duration of intervention. Approximately 74% (36/49) of the studies evaluated participants after using the intervention for 1 to 3 months. About 22% (11/49) of the studies asked users to use the app for more than four months.

Classification using the WHO Classification of Digital Health Interventions [36] revealed that several mental health apps (n=11) exhibited more than one function. Three features that were most commonly found among the evaluated interventions were targeted client communication (n=39), client-to-client communication (n=8), and on-demand information services (n=5). These interventions also targeted a range of mental health–related conditions, with depressive disorders as the most common condition (n=13), followed by interventions targeting participants’ overall well-being (n=11). Other targeted conditions included anxiety disorders (n=2), family violence (n=1), feeding and eating disorders (n=6), neurodevelopmental disorders (n=1), schizophrenia spectrum and other psychotic disorders (n=4), substance use and addictive disorders (n=6). The paper on neurodevelopmental disorders is a study conducted by Backman et al [76] that aims to deliver psychoeducation to adolescents with autism spectrum disorde. Five studies developed interventions that targeted comorbid conditions such as cooccurring depression and alcohol use. 

**Table 1:** Characteristics of included studies (N=49).

Characteristic		Number of studies, n (%)	References
**Type of paper**			
	Full	40 (82)	[39,40,42-46,49-52,54-58,61-63,65-71,73,74,76-87]
	Protocol	9 (18)	[41,47,48,53,59,60,64,72,75]
**Year of publication**			
	2008	1 (2)	[39]
	2009	1 (2)	[40]
	2010	2 (4)	[41,42]
	2011	1 (2)	[43]
	2012	2 (4)	[44,45]
	2013	3 (6)	[46,70,77]
	2014	8 (16)	[47-50,78-81]
	2015	5 (10)	[51-54,87]
	2016	4 (8)	[55,71-73]
	2017	6 (12)	[56-59,82,83]
	2018	12 (25)	[60-63,66-69,74,76,84,86]
	2019	4 (8)	[64,65,75,85]
**Country of study**			
	Australia	12 (25)	[41,42,47,53,56,58,65,67,70,71,73,74]
	Canada	1 (2)	[54]
	Germany	2 (4)	[44,62]
	Ireland	2 (4)	[48,66]
	Japan	1 (2)	[85]
	The Netherlands	3 (6)	[64,75,83]
	New Zealand	1 (2)	[87]
	Norway	1 (2)	[80]
	Romania	1 (2)	[59]
	Spain	1 (2)	[60]
	Sweden	1 (2)	[76]
	United Kingdom	2 (4)	[39,77]
	United States	21 (43)	[40,43,45,46,49-52,55,57,61,63,68,69,72,78,79,81,82,84,86]
**Study sample size**			
	≤100	25 (51)	[40,46,48,50,51,54,55,57-59,61,63,64,68,70-72,75,76,78,79,81-83,85]
	101-1000	17 (35)	[39,42,44,45,47,49,52,56,62,65-67,69,73,84,86,87]
	>1000	6 (12)	[43,53,60,74,77,80]
	Unknown	1 (2)	[41]
**Duration of intervention**			
	≤1 month	10 (20)	[42,47,52,56,61,69,70,73,79,81]
	2-3 months	26 (53)	[39-41,45,48-51,53,54,57-59,62,63,66,67,71,72,75,78,80,83,85-87]
	4-12 months	8 (16)	[44,46,60,64,65,68,76,77]
	>12 months	3 (6)	[55,74,82]
	Unknown	2 (4)	[43,84]
**Type of app^a^**			
	On-demand information services	5 (10)	[56,74,77,85,87]
	Client-to-client communication	8 (16)	[44,48,49,52,70-72,83]
	Personal health tracking	4 (8)	[48,61,65,66]
	Targeted client communication	39 (80)	[39-42,44-48,50-55,57-61,63-73,76,78-84]
	Untargeted client communication (eg, modular delivery)	4 (8)	[43,62,75,86]
**Study design**			
	Randomized controlled trials	38 (78)	[39-41,43-56,58-60,62-65,67-69,72,73,77-84,86]
	Quasi-experimental designs	11 (22)	[42,57,61,66,70,71,74-76,85,87]
**Mental health condition**			
	Anxiety disorders	2 (4)	[41,48]
	Depressive disorders	13 (27)	[40,46,52-55,58,61,71,78,80,82,85]
	Feeding and eating disorders	6 (12)	[44,45,49,51,59,67]
	Other (family violence)	1 (2)	[83]
	Overall well-being	11 (22)	[42,56,57,62,69,74,75,77,79,86,87]
	Schizophrenia spectrum and other psychotic disorders	4 (8)	[50,63,70,72]
	Neurodevelopmental disorders	1 (2)	[76]
	Substance use and addictive disorders	6 (12)	[39,43,60,68,81,84]
	Mixed	5 (10)	[47,64-66,73]

^a^On the basis of World Health Organization Classification of Digital Health Interventions [36]. Some apps report exhibiting multiple functions.

### Analytics Metrics and Analysis

All studies examined usage data in some aspects of their evaluation, and most papers were ascribed to one or more of the following constructs: user adherence [40,46,49-51,53,55,62,64,75,80,82,85], engagement [47,48,52,56,61,65,67,77,79,84], or acceptability [45,57,71]. Although adoption was included in our search, none of the reviewed studies used analytics as a proxy to measure that indicator.

A summary of the findings for analytics metrics and analysis is provided in Table 2. Fifty percent (25/49) of the studies collected 1 or 2 metrics to examine user activity, and 29% (14/49) collected more than four metrics. Most studies had a limited amount of user activity data that were used to explore and understand how participants used their digital interventions.

Across the reviewed studies, different types of metrics were used to evaluate the level of user activity in these interventions. Overall, a heterogeneous selection of metrics was collected, with the number of modules completed (n=27/49) and the session duration (n=24) being the most common metrics. In particular, a few studies collected metrics on the number of times different features were used (n=18). Because these features were highly specific to the intervention being evaluated (ie, number of likes, number of journal entries), these unique metrics were collapsed into a general category called *number of features used*. The *other* category consisted of metrics that did not fall into the common categories and included the bounce rate, the referral source, and the number of characters. The bounce rate and the referral source were collected by 1 of the studies [83] that used Google Analytics [88].

In terms of how metrics were analyzed, many studies [40,42,44-48,50,54,57-59,62,63,65,66,68-70,72-77,79,80,82,85,86] presented usage metrics separately from the main study data using descriptive methods, which included the overall counts, frequencies, means, or SDs. Several studies [39,84] had the main objective of examining how different interventions (eg, behavioral activation) impacted user engagement on the app. These studies conducted analyses using hypothesis testing techniques (eg, chi-square test, *t* tests). Many studies [43,53,60,61,78,81] also attempted to examine if different levels of user activity had a differential effect on the main study outcomes. For example, Paschall et al [43] analyzed reductions in alcohol use by stratifying the course completion rate into 3 categories: high users (70%), medium users (30%-69%), and low users (0%-29%), and reported differential effects within each group. Other studies employed regression analysis and identified varying results. For example, Bidargaddi et al [56] evaluated an app on resource finding and found that the number of log-ins was not associated with the ecological momentary assessment outcomes.

**Table 2:** Types of usage metrics collected in included studies (N=49).

Characteristic		Number of studies, n (%)	References
**Number of usage metrics analyzed**			
	1	17 (35)	[39,41,43,50,56,58-60,67,73,76,77,81,84-87]
	2	8 (16)	[42,45,47,51,54,68,69,80]
	3	10 (20)	[40,44,49,53,62,64,65,72,75,82]
	4+	14 (29)	[46,48,52,55,57,61,63,66,70,71,74,78,79,83]
**Type of metrics collected (multiselect)**			
	Number of clicks	1(2)	[61]
	Number of features used	18 (37)	[40,41,46,48,51,52,57,62,63,65,66,68,69,71,72,74,79,83]
	Number of log-ins	20 (41)	[49,52,54-57,61-64,66,70-72,74,75,79,80,83,87]
	Number of modules	27 (55)	[43,45-47,50,53-55,57,58,60,62,64,66,67,69,70,73-76,78-82,86]
	Number of page views	10 (20)	[44,48,49,51,61,70,74,77,79,83]
	Number of posts	5 (10)	[44,63,70,71,78]
	Number of sessions	9 (18)	[39,42,46,48,52,53,55,74,78]
	Rate of return	4 (8)	[70,71,74,83]
	Session duration	24(49)	[40,42,45,46,48,49,52,53,55,57,61,64-66,68,70,74,75,78,79,82-85]
	Other (eg, calculated metrics)	14 (29)	[40,44,46,47,52,55,59,61,65,70,72,74,82]

Although some studies have also reported similar results [49,51,84], associations between specific analytic measures and outcomes have been identified. For example, Saekow et al [51] noted correlations between the number of log-ins and physical outcome measures of the Eating Disorder Examination Questionnaire. Logsdon et al [61] also found that the duration of a depression intervention was associated with reported attitudes of the participants. Another study on CATCH-IT [78], a module-based intervention for depression, also found that an increased duration on the website was associated with changes in depressive symptoms.

Two studies incorporated more sophisticated metrics. Stallman and Kavanagh [74] leveraged the Google Analytics engine to examine if referral source as a metric influenced their outcomes. They found that different referral sources resulted in different expectations and disparities in user activities. In addition, Schlosser et al [63] separated their user activity data into active use and passive use by calculating the active use rate. The authors [63] defined active engagement as interacting with the features of the app and passive use as logging onto the app but not interacting with a specific feature of the app (ie, posting a moment, completing a challenge, participating in peer interactions). However, their exploration did not find any significant relationships between these user activity metrics and changes in primary and secondary outcomes.

The findings of this scoping review will also be used to inform the selection of analytics metrics to be examined as a potential exploratory component of a larger RCT evaluating an mHealth intervention, Thought Spot [26].

## Discussion

### Principal Findings

This scoping review explores how usage data are characterized and analyzed in evaluations of digital mental health innovations for transition-aged youth. There is an unprecedented demand to address current concerns and gaps in adolescent mental health [89,90], and the increasing ubiquity of mobile apps provides a unique opportunity to do so [91]. The recent nature of the 49 papers included in this review suggests a growing movement toward integrating analytics into mHealth evaluations [25]. In fact, the observed diversity in objectives, sample size, and duration of exposure suggests that analytics can bring value in evaluations of efficacy, effectiveness, and feasibility studies. Analytics is also of particular interest in the mental health domain because of its potential role in digital phenotyping, which is a growing area of study that explores the intersection of behavior and passively collected data [92-94]. As a result, understanding the value and significance of analytics can help to enhance our understanding and applications in digital psychiatry [95]. However, despite the increasing ubiquity of analytics in evaluations, the overall findings of the current review highlight several gaps in evidence [84].

Foremost, there was significant heterogeneity in the construct that analytics was ascribed to. For example, Bidargaddi et al [56] used the term *engagement*, whereas Rickhi et al [54] used the term *program use*, even though both studies used the number of log-ins as their usage metric. Similar variability was also found in a recent review of analytic indicators by Pham et al [25] who suggest that distinctions between certain constructs such as acceptance and engagement are emerging. Likewise, although *engagement* was found to be the most common construct used to describe analytics, it is not surprising because there is a significant body of literature on conceptualizing this term [25,96,97]. As such, the emerging demarcation of these terminologies will likely foster guidance on the role of analytics in measuring these constructs.

In addition, although adoption was included in the search strategy, no studies in our included papers examined adoption specifically and/or used analytics as a proxy to abstract the construct. This is interesting in contrast to literature in clinical informatics where adoption is often an end goal for implementation of technology [98-100] and is well described in implementation frameworks [101]. For example, the clinical adoption metamodel [101] uses the definition from Hall [102] and describes adoption as the process (eg, activities and decisions) of integrating a specific technology into an organization. Similarly, Roger’s diffusion of innovation theory defines adoption as “the decision to make full use of an innovation as the best course of action” [103]. Although *adoption* was a keyword included in our search strategy, we are unable to identify any studies that used analytics to measure adoption. The disparity between the clinical informatics literature and that of consumer informatics literature is intriguing. Increasing awareness of the potential of digital mental health in connecting care and empowering patients to participate in their own care [104] requires users to utilize the technology as appropriate to successfully realize the intended benefits [23]. Addressing this discordance is particularly important, given that Torous et al [105] noted that interest in technology does not necessarily result in the uptake and usage of technology. As such, similar to clinical informatics, it may be valuable to understand and characterize the successful adoption of consumer health information technology [63].

Furthermore, understanding the process of how transition-aged youth decide to adopt technology would provide further insight into the characteristics of successful adoption [23,39,84]. Transition-aged youth are part of the generation where access to the internet and usage of mobile apps are fundamental to their daily lives [106]. As commented by Bewick et al [39], it is unclear how consumers choose to engage and uptake technologies. Although approaches such as user-centered design methodology [107] and gamification [108] have made significant progress in closing the gap between perceived and actual user needs, real-world engagement remains fairly low and heterogeneous [109]. As such, identifying predictors of uptake by understanding how this population adopts these types of technology may help inform the development of an app that will result in adoption by the intended population [49,55,74,80,84].

The other key finding of this review is the significant heterogeneity in the number and types of metrics collected. We observed that most studies collected 1 or 2 metrics and usually included the number of modules completed and the session duration. These findings are in contrast to a similar review by Pham et al [25] on chronic diseases, which found that the number of recorded measures and the frequency of interactions were the most prevalent metrics collected. Given that many studies evaluated computerized cognitive-behavioral therapy programs that are modular in nature, it is not surprising that module completion was most commonly found in this review. Nevertheless, the significance and value of each of these metrics are unclear. At the time of writing this paper, a review by Pham et al [25] attempted to address this issue by categorizing metrics into different components of engagement: amount, breadth, depth, and duration. In addition, Pham et al [25] attempted to provide guidance on how to select appropriate metrics for an evaluation. Although they have made significant progress on some of these issues [25], we believe that the importance and consistency of this issue warrants more in-depth discussion.

The heterogeneity in analytics metrics between studies also generates another level of complexity for analysis. For the various metrics selected, many studies often characterized their analytics findings as constituting high usage [45,50,58,63,72,73,79] or low usage [39,73,80]. However, there does not seem to be a standard as to what constitutes these demarcations [81]. Although it is likely that the boundaries of high or low usage are relative to authors’ expectations, the lack of a standardized definition to demarcate high or low usage can generate confusion. We found that many studies referenced other studies to define high or successful usage levels [50,70,71,81]. However, it is unclear if the threshold used for defining high usage in a single evaluation merits sufficient external validity to be used by others. Several studies also referred to the construct of *dosage* [54,80,81], particularly with respect to if users have received sufficient dosage to reap the benefits of the app. Although usage data may help provide insight into amount of usage, it is unclear how to determine if usage becomes sufficient, insufficient, or too much [61,80-82]. Identifying robust methods to evaluate the complexity of analytic data and understanding how these levels are demarcated to contribute to the validity of the evaluation would be useful.

Similar to the observations made by Pham et al [25], many analyses of analytics in our included studies were limited to descriptive analysis, with only a few studies examining the relationship between analytic metrics and primary outcomes. Although these results were primarily mixed, it is also unclear what significance these correlations had in the overall evaluation. Studies that identified a correlation [78,81] suggested that this finding indicated that usage of the app was beneficial for the specific outcome. In other studies where no relationship was identified between analytics and outcomes, some authors questioned if this finding reflected a lack of efficacy of the mHealth app [56]. A few studies also conducted a per-protocol analysis in addition to the traditional intention-to-treat approach [110]. Although intention-to-treat [110] is the gold standard for RCTs, the high attrition of electronic health (eHealth) solutions may be too conservative in evaluating the actual impact on users who have sustained use of a solution [43].

### Limitations

Several limitations should be noted when interpreting the findings of this scoping review. Because consumer health informatics has become popularized only over the last decade [31], we focused our review on the literature from 2008 onward. Although our findings suggest that most papers have been published within the last 6 years, it is possible that we might have missed papers outside of this window in our search strategy. In addition, due to our scope of work on Thought Spot [26,27,29], our search was limited to examining interventions designed exclusively for transition-aged youth. Because we excluded papers evaluating interventions for other populations (eg, adults) and disease sites (eg, cardiology), there may have been evidence and guidance on the use of analytics in other areas of health care [25]. Evaluating how analytics is applied in other domains of health care may provide more comprehensive insight into the role of analytics in consumer health informatics. It is also interesting to note that most of the papers included in this review were published in North America and Europe, and there was a lack of studies from other countries, including those in Asia and Africa. An evaluation of how adoption and engagement are measured in studies conducted in other countries is warranted.

A second limitation is that, as per guidelines established for scoping reviews [30], we did not evaluate the quality of the included studies. In addition, our review focused on scientific literature and did not include interviews with key stakeholders or a survey of the gray literature. Future research should explore these other sources of literature and evaluate the quality of the studies identified for subsequent systematic reviews.

Finally, it should be noted that, due to the limited number of papers in this scoping review, we did not limit our analyses by mental health conditions and types of research designs. In particular, it would be useful to examine how analytics is used to support the evaluation of different solutions and objectives. Studies such as Torous et al [111] and Connolly et al [112] have identified that individuals with different mental health conditions and culture can impact their engagement with technology. Future work should evaluate how these differences should be explored in the evaluation of adoption and engagement with technology.

### Future Directions

This scoping review provides a preliminary insight into the role of analytics in evaluating mHealth apps for transition-aged youth and identifies both progress that has been made and future areas of exploration. Most importantly, this review adds to the sparse literature on analytics and highlights the need for researchers to assess and standardize how to integrate analytics into their evaluation plans [25]. The addition of more case studies on analytics may help to identify emergent patterns that help us understand how transition-aged youth decide to adopt a solution to address their needs [14].

In addition, several areas of exploration have been identified for researchers. As suggested by Pham et al [25], there is currently no guidance on how to maximize the value of these data. However, in other fields such as marketing, analytics is a robust component in evaluating the success of products and solutions [113]. Thus, it is important to understand how to analyze these findings. This may include knowing how to identify thresholds for high and low users as well as recognizing the significance of correlations and relationships between analytic metrics and primary outcomes. Given that these thresholds may differ between different clinical conditions and methodologies, subanalyses examining how adoption and engagement are explored for different populations (eg, cultures) and research designs may be of interest. Additionally, similar to the progress made in clinical informatics [98-100], understanding the characteristics and predictors of successful adoption of a consumer technology would provide insights into developing a roadmap toward successful mHealth development for transition-aged youth [114].

### Conclusions

This scoping review provided initial insights into the role of analytics in evaluating mobile mental health apps (mHealth) for transition-aged youth. Our analysis of 49 studies published between 2008 and 2019 revealed that the use of analytics is becoming increasingly ubiquitous in evaluating mHealth apps for transition-aged youth. Despite recent progress in the use of analytics, there is still heterogeneity in understanding the significance and value of analytics in these evaluations. In addition, the lack of guidance on metrics selection and analysis warrants future exploration. As digital mental health care continues to grow in popularity, particularly for transition-aged youth, understanding analytics and its impact on evaluations would help to streamline the journey toward using digital interventions to foster better mental health care for this population.

## Appendix

Multimedia Appendix 1 Sample search strategy for scoping review.

Multimedia Appendix 2 Data extraction table for scoping review.

## Abbreviations

eHealth: electronic health
MEDLINE: Medical Literature Analysis and Retrieval System
mHealth: mobile health
RCT: randomized controlled trial
WHO: World Health Organization
