# Supplementary material for: Surveying the Role of Analytics in Evaluating Digital Mental Health Interventions for Transition-Aged Youth: Scoping Review
Source: JMIR Ment Health. 2020 Jun 25;7(6):e15942. doi: 10.2196/15942 (PMC7381002; doi:10.2196/15942)
Supplement: Multimedia Appendix 1 [file mental_v7i6e15942_app1.docx]

DATABASE: MEDLINE DATE CONDUCTED: JULY 2018

| **#** | **Searches** | **Results** | **Annotations** |
| --- | --- | --- | --- |
| 1 | (uptake or accept* or adopt* or engag* or "use" or usage or adherence or compliance).kf,tw. | 3524610 | adoption terms |
| 2 | (efficacy or evaluat* or trial).kf,tw. | 3781699 | evaluation mhealth terms |
| 3 | exp TELEMEDICINE/ | 22954 | MeSH for mhealth explode |
| 4 | (ehealth or e-health or mhealth or m-health or telemedicine or tele-medicine or telehealth or tele-health).kf,tw. | 18402 | mhealth terms |
| 5 | ((mobile or internet or internet-based or "internet based" or web-based or web or "web based" or online) adj2 (intervention or program)).kf,tw. | 3642 | mhealth terms |
| 6 | exp Young Adult/ | 668760 | YA population |
| 7 | exp ADOLESCENT/ | 1869163 | YA population |
| 8 | (youth or young adult* or "emerg* adult" or "transition-aged youth" or "transition aged youth" or adolescen* or university student* or college student* or young people*).kf,tw. | 394052 | YA population |
| 9 | exp Mental Health/ | 31086 | mental health terms |
| 10 | exp Mental Disorders/ | 1124842 | Mental health terms |
| 11 | (mental health or mental disorder* or mental well* or addict* or psychological health or depression or anxiety or mood or obsessive compulsive disorder).kf,tw. | 600445 | Mental health terms |
| 12 | (depress: or behav:).mp. or exp mental disorders/ or psych:.mp. | 3503939 | ***NOT USED*** |
| 13 | (psych* or behav*).kf,tw. | 1771103 | ***NOT USED*** |
| 14 | 1 or 2 | 6427181 | adoption and evaluation combined |
| 15 | 3 or 4 or 5 | 34054 | mhealth or ehealth combined |
| 16 | 6 or 7 or 8 | 2306683 | transition-aged youth combined |
| 17 | 9 or 10 or 11 | 1472296 | mental health combined |
| 18 | 14 and 15 and 16 and 17 | 840 | Combined |
| 19 | limit 18 to (english language and yr="2008 -Current") | 763 | Limiters Added |
